# Perceptual confidence judgments reflect self-consistency

**DOI:** 10.1167/jov.21.12.8

**Published:** 2021-11-18

**Authors:** Baptiste Caziot, Pascal Mamassian

**Affiliations:** 1 Laboratoire des Systèmes Perceptifs (CNS UMR 8248), DEC, ENS, PSL University, Paris, France; 2 Laboratoire des Systèmes Perceptifs (CNS UMR 8248), DEC, ENS, PSL University, Paris, France

**Keywords:** confidence, decisions, response times, perceptual biases, after-effects, priors

## Abstract

Each perceptual decision is commonly attached to a judgment of confidence in the uncertainty of that decision. Confidence is classically defined as the estimate of the posterior probability of the decision to be correct, given the evidence. Here we argue that correctness is neither a valid normative statement of what observers should be doing after their perceptual decision nor a proper descriptive statement of what they actually do. Instead, we propose that perceivers aim at being self-consistent with themselves. We present behavioral evidence obtained in two separate psychophysical experiments that human observers achieve that aim. In one experiment adaptation led to aftereffects, and in the other prior stimulus occurrences were manipulated. We show that confidence judgments perfectly follow changes in perceptual reports and response times, regardless of the nature of the bias. Although observers are able to judge the validity of their percepts, they are oblivious to how biased these percepts are. Focusing on self-consistency rather than correctness leads us to interpret confidence as an estimate of the reliability of one's perceptual decision rather than a distance to an unattainable truth.

## Introduction

An observer trying to perceive objects in her environment must infer them from noisy sensory measurements (Mamassian, Landy, & Maloney, 2002). This perceptual inference is informed by a combination of noisy measurements in an environment that can fluctuate (for example, because of differences in illumination at noon and dusk) and by some prior beliefs of the observer that sometimes can be updated (Gold & Stocker, 2017; Knill & Richards, 1996; Mamassian et al., 2002; Weiss, Simoncelli, & Adelson, 2002). The observer can subsequently make decisions by applying a decision rule on the inferred variables. These decisions are—at least in humans—accompanied by a sense of uncertainty called confidence. It remains unclear how this sense of confidence is computed and how it relates to the fluctuations of the environment and to prior beliefs. Confidence is classically defined as the posterior probability of a decision to be correct, given the evidence (Drugowitsch, Moreno-Bote, & Pouget, 2014; Hangya, Sanders, & Kepecs, 2016; Meyniel, Sigman, & Mainen, 2015; Pouget, Drugowitsch, & Kepecs, 2016; Sanders, Hangya, & Kepecs, 2016). Here, we argue that this definition cannot withstand close examination. Correctness implies agreement with the true state of the world—which observers do not know—thus dismissing inherent biases of the perceptual system and the role of prior beliefs.

If the observer cannot use the true state of the world as the reference against which she is trying to compare her perceptual decision, what can she use? It might be tempting to replace the objective truth by the subjective percept of the observer, but this leads to circular reasoning. It is indeed rare that an observer dismisses a decision she just took, because if she thinks her decision is wrong, why did she take it in the first place? One way out of this circularity is to assume that the observer is considering not just the interpretation corresponding to her percept but also multiple alternative interpretations. Although one interpretation is eventually chosen, the observer may also have access to some information about alternative interpretations that have been discarded. The clearest description of such a theory is the balance of evidence model proposed by Vickers (1979). In this model, each possible interpretation is described by a race (i.e., an accumulation of sensory evidence), the perceptual decision is the winning race, and the balance of evidence is the evidence of the losing race at the time of the perceptual decision. Although this model is very powerful to account for experiments run in the laboratory where the number of alternative interpretations is usually small and known to the observer, it is more difficult to see how it could generalize to more natural situations where the number of possible interpretations each time we glance around is literally infinite.

**Figure 1.**
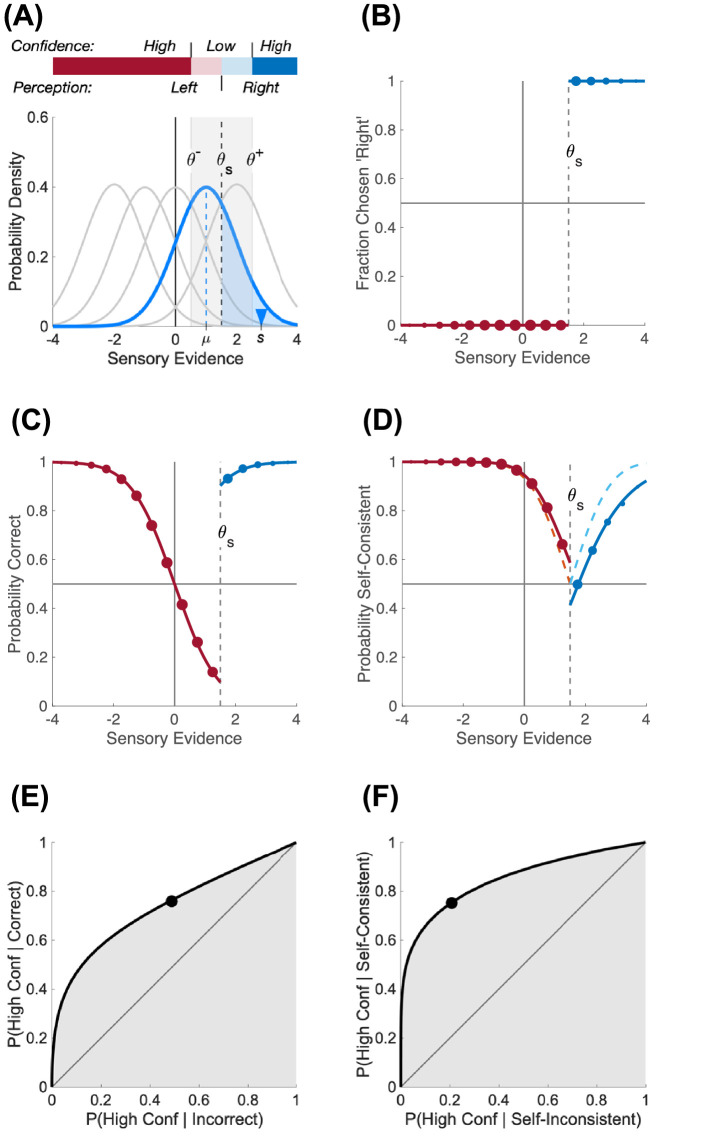
Difference between correctness and self-consistency. (A) Simulation of an experiment where one of five oriented stimuli can be presented, where orientations to the right of vertical are coded as positive. Because of sensory noise, each stimulus generates an internal representation whose uncertainty can be represented by a distribution of sensory evidence (shown as one of five Gaussian distributions). On each trial, the observer has to make an inference on the stimulus based on a sample ***S*** from the corresponding distribution (blue triangle). A perceptual decision is taken as to whether the stimulus is tilted to the right or to the left by comparing this sample of sensory evidence to a subjective criterion θ*_s_*. If confidence evidence matches sensory evidence, high and low confidence judgments can also be taken on this internal representation by comparing the evidence to two new criteria (θ^+^ and θ^–^) on either side of the subjective criterion. (B) Fraction of chosen “right” response. The sensory evidence is binned, and dot size is proportional to the number of trials in that bin. According to the perceptual decision rule, any value of the sensory evidence larger than the subjective criterion leads to a “right” response (shown in blue), and any value below leads to a “left” response (red). (C) Probability correct. Physical stimuli were tilted to the right when their stimulus strength (indicated by the mean, µ, of the uncertainty distribution) was positive. Probability correct is at chance when the sensory evidence is at the objective criterion (zero) and is presenting a sharp discontinuity at the subjective criterion. (D) Probability self-consistent. Self-consistency represents the probability of responding the same thing if the same physical stimulus is presented. Probability self-consistent is almost symmetric about the subjective criterion (binned dots of sensory evidence and solid line) and is exactly symmetric if any stimulus strength (instead of 5) was presented with uniform probability (dashed line). (E) Type II ROC for correctness. The Type II ROC plots the conditional probability of reporting high confidence when the observer is correct as a function of the probability of reporting high confidence when the observer is incorrect. The black dot corresponds to the particular choice of criteria θ^+^ and θ^–^ in (A) and can be plotted by the experimenter based on the participant's Type I and Type II responses. The smooth curve is obtained by simulating different confidence criteria. The area under the Type II ROC curve is a measure of confidence sensitivity. (F) Type II ROC for self-consistency. Same as in (E) but correctness is replaced by self-consistency. The area under the curve is larger, indicating an actual better confidence sensitivity when the perceptual bias is taken into account.

Instead of considering that confidence refers to the distance to the true state of the world, or that the observer has access to multiple interpretations compatible with some sensory evidence, we propose here that perceptual confidence is a measure of the stability of the perceptual decision given the sensory evidence. More specifically, we hypothesize that confidence judgments reflect an evaluation of the extent to which the current perceptual decision is self-consistent with other decisions the observer could have taken, previously or in the future, in the same conditions. In other words, self-consistency is a measure of reproducibility of perceptual decisions. If the same stimulus was presented to an observer in the exact same experimental conditions, high self-consistency corresponds to large probability of perceiving the same thing. In contrast, correctness is a measure of matching of perceptual decisions with the true state of the world. Self-consistency departs from correctness as soon as observers adopt a subjective sensory criterion that differs from the objective criterion that separates physical object categories (Figure 1; see also Supplementary Material S1).

As an illustration, Figure 1 shows a simulated experiment where one of five oriented stimuli can be presented to an observer. Her task is to estimate whether the presented stimulus is tilted to the right or to the left. To do so, she might rely on some sensory evidence extracted from the presented stimulus and use an internal criterion to decide whether the sensory evidence is more likely coming from a right- or left-tilted stimulus. An optimal observer would place her sensory criterion at 0 (vertical), but the observer might be biased in setting her criterion (Figure 1A). If the observer is using the same sensory evidence to estimate her confidence about the validity of her perceptual decision (for alternative models, see Mamassian & de Gardelle, 2021), two confidence criteria can be placed on either side of the sensory criterion so as to generate confidence ratings on two levels (low when the evidence is close to the sensory criterion and high otherwise). A biased sensory criterion to the right of vertical creates an inflation of left orientation decisions (Figure 1B). As a consequence, the trials where the sensory evidence lies in between the objective sensory criterion (at 0) and the subjective criterion (at θ*_s_*) are likely to be incorrect (Figure 1C). In contrast, these trials will still be mostly self-consistent with past similar trials (Figure 1D). This difference between correctness and self-consistency has an impact on the estimated confidence sensitivity. Confidence sensitivity can be measured as the area under the Type II receiving operating characteristic (ROC) curve that plots the Type II hit against false alarm rates. The Type II hit rate is usually defined as the conditional probability of reporting a high-confidence judgment given that the perceptual decision was correct, and the Type II false alarm rate is the conditional probability of reporting a high-confidence judgment given that the perceptual decision was incorrect (Figure 1E). Replacing correctness by self-consistency changes the Type II ROC curve (Figure 1F) and therefore the estimated confidence sensitivity. Although correctness is well defined and controlled by the experimenter, self-consistency can only be approximated. The experimenter does not have access to the internal sensory criterion used by the observer, and this criterion may also be subject to noise and other factors such as asymmetrical rewards. However, the experimenter has access to the Type I results that give for each stimulus strength the fraction of perceptual responses for each perceptual category (here, left and right). The self-consistent decision for any stimulus strength can then be assumed to be the most frequent one (see Supplementary Material S1). Therefore, from the experimenter's perspective, one can place as many points on the Type II ROC curve as there are confidence criteria (1 point here for a high vs. low confidence judgment, or 3 points for confidence judged on a 4-point rating scale). In our simulations, using correctness instead of self-consistency would lead the experimenter to report a reduced confidence sensitivity.

**Figure 2.**
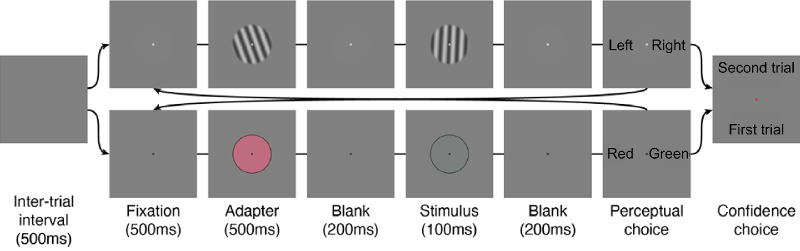
Depiction of a trial sequence in Experiment 1. Trials started with a 500 ms blank screen. Then, alternating from block to block, observers started with an orientation (or color) discrimination task. An adapter stimulus was displayed for 500 ms, followed by a 200 ms blank, followed by a 100 ms test stimulus. After another 200 ms blank, a response screen was displayed prompting observers to report the perceived orientation (or color) of the stimulus. Following the first perceptual response, the second task started with an identical sequence. After the observer reported the perceived color (or orientation) for the second task, a new response screen appeared prompting them to report which of their two preceding responses they felt more confident about having answered correctly.

To understand the implications of self-consistency for confidence judgments, we conducted two psychophysical experiments. The two experiments are based on the same basic procedure and were designed to test whether the relationships among observers’ sensitivity, response times, and confidence judgments are preserved when the percepts of the observers are systematically biased. The experiments differed in the way these biases were generated, either at a low level of perceptual processing with sensory adaptation and the resulting after-effects or at a higher level with biased prior probability of stimuli and the resulting expectations of the observer. Rigorous measurements of confidence judgments are notoriously difficult to accomplish (Clarke, Birdsall, & Tanner, 1959; Fleming & Lau, 2014). We adopted the criterion-free confidence forced-choice procedure initiated by Barthelmé and Mamassian (2009; de Gardelle & Mamassian, 2014). In this procedure, observers are forced to choose which of two perceptual decisions they just provided they believe to be more likely to be correct. In an effort to prevent observers from comparing the perceived intensity of the two stimuli, instead of the correctness of their answer, we asked observers to compare their confidence across two different visual dimensions (orientation and color). In previous work, we have shown that observers can judge confidence across tasks as well as within the same task (de Gardelle & Mamassian, 2014). On each confidence pair trial (Figure 2), observers had to perform a pair of perceptual decisions—an orientation discrimination task and a color discrimination task—and then report which one of their perceptual decisions they thought was most likely to be correct. In these instructions given to the participants, correctness refers to the subjective impression of being correct, not necessarily to the objective truth. For the confidence judgments, each stimulus condition in one task was compared to each stimulus condition in the other task. This carefully controlled experimental design has two main advantages: (1) the observers’ confidence judgments cannot be confounded by low-level sensory signals, as they must base their judgments on the validity of their decision in two different tasks; and (2) their confidence judgments are not contaminated by confidence criterion effects.

As a preview of our results, we demonstrate that observers’ sense of confidence matched their perceptual reports, not the stimulus. In the first experiment, where we induced low-level biases through sensory adaptation and after-effects, the mapping between perceptual reports and confidence judgments remained perfectly identical across biases. In the second experiment, where we induced perceptual biases by manipulating the prior probability of occurrence of stimuli, the mapping between confidence and perceptual reports was here again perfectly preserved across the observers’ biases. Overall, these results are strong evidence that observers’ sense of confidence does not match an appreciation that their perceptual decision is correct. Instead, confidence matches the observers’ subjective perceptual experiences, regardless of objective truth. Therefore, our results are in agreement with a framework where confidence is an estimate of self-consistency.

## Materials and methods

### Participants and apparatus

Observers were 16 students and faculty (mean age ± *SD*, 26.9 ± 4.2 years), including one author, with normal or correct-to-normal vision. All were experienced observers, and all but the author were naive to the purpose of the experiment. Half of the observers ran the “after-effect” experiment first, and half started with the “prior” experiment. Stimuli were displayed on a Sony Trinitron CRT GDM-F520 (Sony Corporation, Tokyo, Japan) at 57.3 cm, with three color channels linearized independently with a Minolta CS-100A Chroma Meter (Konica Minolta, Tokyo, Japan). The monitor midpoint level on all three guns (*xyY* = 0.278, 0.307, 60.3) was used as a neutral gray reference after 5 minutes of adaptation. Colors were generated from a relative Derrington–Krauskopf–Lennie (DKL) color space (Derrington, Krauskopf, & Lennie, 1984) and then converted to red, green, and blue (RGB) values using standard procedures (Zaidi & Halevy, 1993). Colors were modulated on the L–M axis (“red–green” axis) because, unlike the other two color axes of the DKL color space, response times are symmetrical about the neutral point (Wool, Komban, Kremkow, Jansen, Li, Alonso, & Zaidi, 2015). Color units are fractions of the maximum saturation that could be displayed on the monitor along the L–M axis (*xyY* = 0.320, 0.287, 59.8 and *xyY* = 0.207, 0.325, 55.5) while approximately on the same equiluminant plane as the neutral gray and with the sign indicative of the direction (such that greens are negative). In other words, a color value of −0.1 means that the monitor displayed a green color that had the same luminance as the background and had 10% of the maximum saturation that the monitor was able to display; similarly for a value of +0.1, but with a red hue.

### Stimulus and procedure

Observers alternated between two tasks. The first task was a grating orientation discrimination. A 10° grating (2 cycles per degree [cpd], 44% contrast, random phase, and Hann window) was tilted by one of seven values between −2.1° and +2.1° (left to right) relative to the vertical in the “after-effect” experiment. The “prior” experiment used one of seven values ranging from −4° to +2° relative to the vertical in the “left” blocks and from −2 to +4 relative to the vertical in the “right” blocks. (The spatial frequency of the stimulus was lowered to 0.5 cpd in Figure 2 for clarity.) Observers were instructed to report whether the top of the grating was more to the left (counterclockwise) or to the right (clockwise) of vertical by pressing the left and right arrow keys on a keyboard. The second task was a color discrimination task with a 10° color patch of one of seven values between −0.15 and +0.15 maximum gamut (green to red, see above) surrounded by a high-contrast black contour (7 arcmin, 97% contrast) in the “after-effect” experiment. In the “prior” experiment, colored stimuli consisted of pixels (16 arcmin) whose hues were normally distributed with a standard deviation of 0.1, across a mean varying from −0.06 to +0.03 in “green” blocks and −0.03 to +0.06 in “red” blocks (see Supplementary Material S2, Figure S1). Observers had to report whether the colored patch was green or red relative to the background by pressing the same two arrow keys as for the orientation task.

To induce adaptation in the first experiment, a brief adapter was displayed prior to the test stimulus. In the orientation task, the adapter grating was oriented −20°, 0°, or +20°, with a phase randomly changing every 50 ms to prevent luminance adaptation. In the color discrimination task, the adapter had a value of −0.75, 0 or +0.75. Orientation and color adapters were completely randomized within a block of trials (i.e., any of the three orientation and three color adapters could be presented in a trial within a block).

The trial sequence for the “after-effect” experiment was as follows (see Figure 2A): After 500 ms fixation, an adapter was displayed for 500 ms; after a 200 ms blank, the test stimulus was displayed for 100 ms. Finally, after another 200 ms blank, a response screen was displayed until response. The response screen had the words “left” and “right” displayed 8° to the right and left of fixation in the orientation discrimination task and the words “red” and “green” in the color discrimination task. (These words have been enlarged in Figure 2 for clarity.) This was to make sure the observers would not forget the response mapping of colors and direction arrows, but all observers reported having no difficulties in learning that mapping after a few training trials.

After observers performed a pair of trials (one for each task), another response screen was displayed 200 ms after their last response. This response screen contained the words “first” and “second” at 8° above and below fixation. Observers were instructed to report whether they felt more confident in having answered correctly in the first or second task of the trials pair by pressing the up or down arrows of the keyboard. Then, the next trial started after a 500 ms blank screen. The trial sequence was the same in the “prior” experiment, except that there was no adapter (500 ms fixation, 100 ms stimulus, 200 ms blank, response screen). Observers reported no difficulties in following the trial sequence. No feedback was provided.

The “after-effect” experiment consisted of 882 trials (each with two perceptual responses and one confidence judgment)—one trial for each combination of orientation adapters, orientation test angles, color adapters, color test values, and task order (3 × 7 × 3 × 7 × 2 = 882). The task order was kept constant within each of the 16 blocks of 63 trials and alternated from block to block, counterbalanced across observers. The entire experiment lasted between 75 and 90 minutes. The “prior” experiment consisted of 784 trials—two trials for each combination of the two orientation ranges of seven values, the two color ranges of seven, and two task orders (2 × 2 × 7 × 2 × 7 × 2 = 784). The four combinations of orientation and color ranges (O_1_C_1_, O_2_C_1_, O_1_C_2_, O_2_C_2_) were counterbalanced across observers using a Latin square design where the first- and second-order probabilities were counterbalanced. For each combination the observers ran two successive blocks (eight blocks in total) of 98 trials with different task order (orientation/color) on each block, counterbalanced across observers.

### Analyses

We recorded three behavioral variables: perceptual reports (left/right or green/red), response times (time of perceptual responses since stimulus onset), and confidence judgments (higher or lower confidence than the other trial of the pair). We fitted curves to the data for each observer individually by finding the set of parameters that maximized the likelihood of the model given observed behavior. This required fitting all three variables at the same time by combining the likelihoods. The likelihoods of the perceptual reports and confidence judgments are given by a binomial distribution. Computing the likelihood of the response times was more intricate, and to a first approximation we assumed that it followed a normal distribution. We estimated the variance of this distribution by bootstrapping the response time in each condition and used the standard deviation of the distribution of resampled medians (which followed approximately a normal distribution). Moreover, given the number of fitted curves (16 observers × 2 tasks × 3 adapters × 3 metrics = 288 in total) some fits inevitably converged poorly. To prevent bad fits, we used a quasi-Newtonian gradient descent with reasonable constraints on the possible parameters: Response times could not be negative, ratios of confidence judgments could not be greater than 1 or lower than 0, and biases and adaptation strengths could not be greater than twice the range of tested values.

Perceptual decisions were fitted with cumulative normal functions with three parameters (see Equation 1): an overall bias (µ*_p_*; the value of the point of subjective equality [PSE] for the neutral adapter), the amplitude (α*_p_*) of the after-effect (how much the PSEs are shifted, symmetrically, by the adapter), and the standard deviation (σ*_p_*) of sensory noise (reflecting the inverse of observer sensitivity):

(1) $$\hat{P}=F\left(S,\mu_{p}+\alpha_{p}A,\sigma_{p}^{2}\right)$$

where $\hat{P}$ is the predicted probability to report the stimulus as “right of vertical” or “red,” *F* is the cumulative normal function, *S* is the stimulus value, and *A* is the adapter value. To compare adaptation strengths and biases in the PSEs across observers, stimuli, and experiments, the amplitude of the after-effect was normalized by the observer's sensitivity for the perceptual decision: α′_*p*_ = α_*p*_/σ_*p*_, and is thus expressed in units of sigma (σ*_p_*).

Response times were fitted with scaled normal probability density functions. These functions capture a peak response time when the stimulus is most ambiguous, and a gradual decrease with the intensity of the stimulus toward a baseline. These functions have five parameters (see Equation 2): residual latency (*R*_0_; baseline response time), response time amplitude (*R_A_*; difference between peak response time and baseline), and, similarly to the perceptual decisions analysis, a general bias (µ*_r_*), an adaptation strength (α*_r_*), and a standard deviation (σ*_r_*) corresponding to the width of the function (how quickly response times decrease):

(2) $$\;\;\;\begin{array}{c} \hat{R}=R_{0}+\left(R_{A}-R_{0}\right)\cdot f\left(S,\mu_{r}+\alpha_{r}A,\sigma_{r}^{2}\right)/f\left(0,0,\sigma_{r}^{2}\right) \end{array}$$

where $\hat{R}$ is the predicted response time, *f* is the normal probability density function, *S* is the stimulus value, and *A* is the adapter value. The denominator is just a constant to restrict the range of the function between *R*_0_ and *R_a_*. The choice of this specific function to fit response times is arbitrary, but it captured data well. To compare biases and after-effect amplitudes with perceptual reports, we also normalized these parameters by the observers’ sensitivity in the perceptual decision: α′_*r*_ = α_*r*_/σ_*p*_.

Confidence choices were fitted by upside-down normal probability density functions, one for the unadapted condition and two for the adapted conditions. Each condition in each task was compared to all conditions in the other task. This let us define a confidence variable as the fraction of times the observer reported being more confident in one condition as compared with all other conditions in the other task. These functions had five parameters (see Equation 3): maximum confidence (*C_max_*) and minimum confidence (*C_min_*) to account for possible individual preferences for one modality above the other and, similarly to perceptual decisions and response times analyses, a general bias (µ*_r_*), an adaptation strength (α*_c_*), and a standard deviation (σ*_r_*) corresponding to the width of the function:

(3) $$\;\;\;\;\;\;\;\;\;\begin{array}{c} \hat{C}=C_{max}-\left(C_{max}-C_{min}\right)\cdot f\left(S,\mu_{c}+\alpha_{c}A,\sigma_{c}\right)/f\left(0,0,\sigma_{c}\right) \end{array}$$

where $\hat{C}$ is the predicted probability to report the trial as confident, *f* is the normal probability density function, *S* is the stimulus value, and *A* is the adapter value. This specific function is also arbitrary but captured data well. Here, again, we normalized the bias and adaptation parameters by the observer sensitivity in the perceptual decision: α′_*c*_ = α_*c*_/σ_*p*_.

For each experiment and task, we analyzed the similarity of the effects of adaptation on three metrics: perceptual decisions (*P*), response times (*R*), and confidence (*C*). For this analysis, we first collected normalized biases and adaptation terms across the 16 observers:

(4) $$\;\;\;\;\;\;\;\;\;\;\begin{array}{c} A_{p}=\left[\begin{array}{c} {\alpha^{'}}_{p1}\\ \vdots \\ {\alpha^{'}}_{p16} \end{array}\right],\;A_{r}=\left[\begin{array}{c} {\alpha^{'}}_{r1}\\ \vdots \\ {\alpha^{'}}_{r16} \end{array}\right],\;A_{c}=\left[\begin{array}{c} {\alpha^{'}}_{c1}\\ \vdots \\ {\alpha^{'}}_{c16} \end{array}\right],\;X=\left[A_{p},A_{r},A_{c}\right] \end{array}$$

where *A_x_* is the vector of normalized adaptations for one metric. We then computed correlations between normalized biases and adaptation terms across the 16 observers:

(5) $$\begin{array}{c} \rho \left(x,y\right)=\frac{{\left(A_{x}-\bar{A_{x}}\right)}^{T}\left(A_{y}-\bar{A_{y}}\right)}{\sqrt{{A_{x}}^{T}A_{x}}\sqrt{{A_{y}}^{T}A_{y}}}\; \end{array}$$

where ρ(*x*,*y*) is the correlation between variables *x* and *y*. We also performed a principal component analysis and extracted the principal components and their associated variance:

(6) $$\begin{array}{c} w_{\left(1\right)}=max\left\{w^{T}X^{T}Xw\right\}\; \end{array}$$

(7) $$\begin{array}{c} \Sigma =E\left[\left(X-E\left[X\right]\right){\left(X-E\left[X\right]\right)}^{T}\right]\; \end{array}$$

where *w*_(1)_ is the first principal component, and Σ is the variance explained by this component.

For model comparisons, we fitted nested models to the data and computed the log-likelihood (*L*) of each model. To do so, we computed the likelihood of each perceptual response, response time, and confidence report given the predicted $\hat{P}$, $\hat{R}$, and $\hat{C}$ of each model as described above. The overall log-likelihood is the sum of the log-likelihood of each metric on each trial. Because the models were nested, adding new parameters should only improve the fits (in practice, fitting errors sometimes cause a model with fewer parameters to fit better, although very rarely). The purpose of this analysis is to estimate whether the increase in model complexity can be justified by the improvement in the goodness of fit (estimated through the likelihood). We used the Akaike Information Criterion [AIC] (Akaike, 1974) where the likelihood of the models is penalized by the number of parameters:

(8) $$\begin{array}{c} AIC=2k-2L\; \end{array}$$

where *k* is the number of parameters, and *L* is the overall log-likelihood of the model. The model with the lowest AIC score is considered to explain the data best. The simplest model has seven parameters: slope of the psychometric function, width of the response times function, response time baseline and peak, width of the confidence function, and confidence maximum and minimum values (µ*_p_* = µ*_r_* = µ*_c_* = 0 and α*_p_* = α*_r_* = α*_c_* = 0). In this model, it is assumed that the observers have no bias and there is no effect of the adapter. The full model has 13 parameters and is essentially identical to the fitted functions aforementioned: the biases and adaptation strength are assumed to be independent for the three sets of curves (µ*_p_* ≠ µ*_r_* ≠ µ*_c_* and α*_p_* ≠ α*_r_* ≠ α*_c_*).

To compute the role of objective and subjective sensory distances to confidence judgments, we regressed the observers’ confidence judgments to the difference in objective and subjective sensory distances between the trials of a pair. Because these metrics are correlated, we used a multivariate probit model to isolate the effect of each variable. We computed sensory distances as follows:

(9) $$\begin{array}{c} d_{O,c}=\frac{S_{c}}{\sigma_{c}}\;\mathrm{and}\;d_{S,c}=\frac{S_{c}-\mu_{c}-a_{c}}{\sigma_{c}}\; \end{array}$$

where *d_O_*_,_*_c_* is the objective sensory distance in the color task, taking into account the stimulus value (*S_c_*) normalized by the observer's sensitivity (σ*_c_*). Likewise, *d_S_*_,_*_c_* is the subjective sensory distance, but this time taking into account the observer's initial bias (µ*_c_*) and adaptation strength (*a_c_*). We then computed the difference in sensory distances for each trial pair:

(10) $$\begin{array}{c} \Delta_{O}=d_{O,o}-d_{O,c}\;\mathrm{and}\;\Delta_{S}=d_{S,o}-d_{S,c}\; \end{array}$$

where *d_O_*_,_*_o_* and *d_O_*_,_*_c_* are the objective sensory distance in the orientation and color tasks, respectively; similarly for *d_S_*_,_*_o_* and *d_S_*_,_*_c_* for the subjective sensory distance. Finally, we fitted a multivariate probit model to the observer's confidence choices using maximum likelihood estimation:

(11) $$\begin{array}{c} p\left(o\right)=\Phi \left(\beta_{0}+\beta_{1}\Delta_{O}+\beta_{2}\Delta_{S}\right)\; \end{array}$$

where *p*(*o*) is the probability to report higher confidence in the response to the orientation discrimination task (rather than the color task), and Φ is the probit linking function.

## Results

### Biases induced by perceptual after-effects

**Figure 3.**
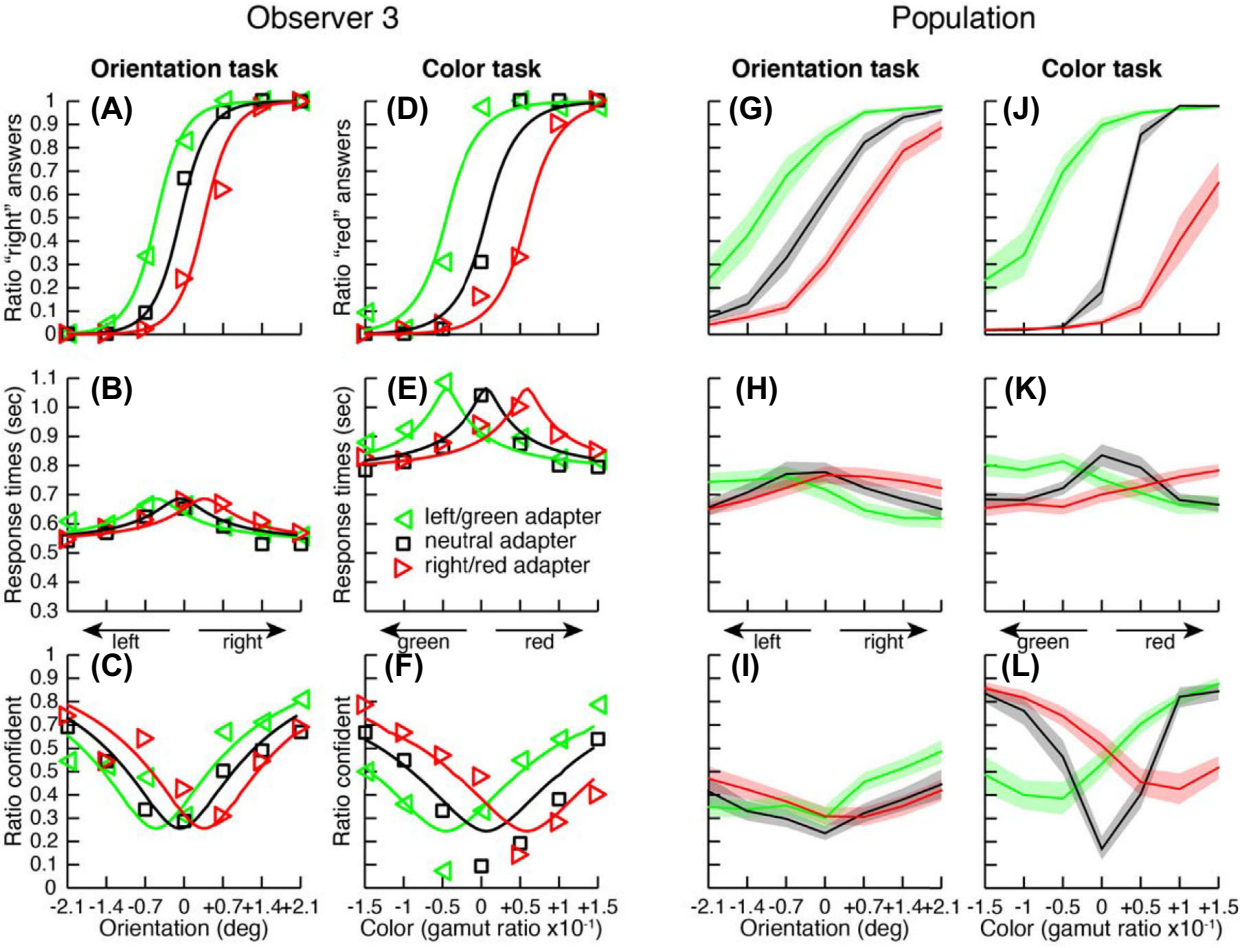
Results for Experiment 1. (A–L) Results from the after-effect experiment for one observer (A–F) and for the population (G–L, *N* = 16). The first column (A–C) depicts the results for the orientation task: (A) Proportion of “right” responses (markers) as a function of grating orientation (abscissa) for the three adapter orientations (markers color) together with predictions from a model implementing confidence as the distance between two competing accumulators (lines; see Supplementary Material S7). (B) Median response times of the perceptual decision. (C) Proportion of perceptual decisions judged more confident than the other perceptual decision. The second column (D–F) depicts the results for the color discrimination task in the same format (replacing “red” for “right” decisions). Population results (G–L) are depicted in the same format as the individual observer. The solid lines represent the mean data and the shaded areas the standard errors across observers.

In the first experiment, we briefly displayed an adapter stimulus before the test stimulus (see Figure 3), inducing tilt after-effects in the orientation task (Campbell & Maffei, 1971; Gibson, 1933; Sekuler & Littlejohn, 1974) and color after-effects in the color task (Troland, 1930; von Kries, 1970). Figure 3A plots the proportion of stimuli perceived by one observer as oriented to the “right” (clockwise) as a function of stimulus orientation (abscissa) and adapter angle. When the test stimulus was preceded by an adapter strongly tilted to the left (−20°), the observer tended to report the test stimulus as tilted to the right more often. For the stimulus to be reported as tilted to the right half the time (PSE, the physical grating orientation that appears vertical), the stimulus had to be physically oriented to the left—that is, in the direction of the adapter orientation. The opposite was true for the adapter oriented to the right (+20°). Therefore, despite the brief duration of the adapter (500 ms), this observer exhibited a clear negative after-effect. We found similar results in the color discrimination task (Figure 3D) and for all observers (Figures 3G and 3J).

Figures 3B and 3E show median response times in the orientation and color discrimination tasks for the same observer as in Figures 3A and 3D (chronometric functions). As expected, the observer was slower when the test value was closer to the PSE (when the stimulus was more difficult to discriminate). This effect around the PSE was present for both neutral and non-neutral adapters. Therefore, the adapter sped up responses when it made the test stimulus perceptually more discriminable and slowed down perceptual decisions when it made it less discriminable, even though the test stimuli remained physically identical. Similar effects were found across all observers (Figures 3H and 3L).

Presenting a brief adapter before a test stimulus produced comparable effects across the psychometric, chronometric, and confidence functions. We established this similarity of the effects with multiple analyses. First, we estimated the strength of adaptation (how much the curves were shifted by the adapters) by fitting curves to the perceptual decisions (see Equation 1), response times (Equation 2), and confidence choices (Equation 3) for each participant individually (see Methods for details). In order to compare adaptation strength across tasks and observers we then normalized all adaptation parameters by the observers’ sensitivities (the slope of their psychometric functions). In the orientation task, the mean normalized adaptation strengths were 0.80, 0.95, and 0.89 for perceptual decisions, response times, and confidence choices, respectively. In the color orientation task, normalized adaptation strengths were 2.14, 2.00, and 1.78, respectively. All adaptations were significantly larger than 0 in a one-tailed *t*-test (see Supplementary Material S3, Table S1) but were not different across metrics in a paired two-tailed *t*-test (see Supplementary Material S3, Tables S2 and S3).

In a second analysis, we reasoned that if adaptation had a comparable effect on perceptual decisions, response times, and confidence judgments, then all three biases should be highly correlated (see Equation 5). This is indeed the case, with pairwise correlations between 0.8 and 1.0 (see Supplementary Material S3, Figure S2 and Tables S4 and S5). This was true for both the initial biases (PSEs for the neutral adapter condition) and the adaptation biases (PSEs for non-neutral adapters). A principal component analysis on all three metrics at the same time (see Equations 6 and 7 in Methods) showed that a main component explained 87% and 91% of the variance for the initial biases and adaptation biases, respectively, which is extremely high.

In a final analysis, we fitted curves to the perceptual decisions, response times, and confidence choices with common parameters between the curves (e.g., the same bias in the PSE). We used model comparison on these nested models to identify which parameters should be included as justified by improvement in the overall likelihood of the model (see Equation 8 in Methods and Supplementary Material S5, Figure S4). This analysis showed that including a common bias in the PSE and a common adaptation parameter for all three variables improved the fits dramatically. But, including separate initial biases for the different variables or separate adaptation factors did not improve fits enough to justify making the model more complex.

In summary, in the first experiment, which used adaption, the observers’ percepts were clearly modified by the adapters despite their brevity. The adapters also impacted response times and confidence judgments despite their volatility from trial to trial. We found no evidence of dissociation among perceptual reports, response times, or confidence judgments. The mapping between these variables was remarkably well preserved across perceptual biases (after-effects), as well as biases in the PSEs. In other words, the single best predictor for confidence—and response times—was the perceptual distance to the PSE.

### Biases induced by prior probabilities

Short-term adaptation is not the only phenomenon that can generate perceptual biases. In a second experiment, we induced biases by changing the prior probability of occurrence of stimuli in different blocks of trials. We took advantage of the central tendency bias (Ashourian & Loewenstein, 2011; Hollingworth, 1910; Huttenlocher, Hedges, & Vevea, 2000; Olkkonen, McCarthy, & Allred, 2014), where trials are reported as closer to the mean stimulus value than they physically are. Within blocks, stimulus intensities were sampled from a range of values not centered on the reference (vertical orientation or neutral gray color). The procedure was identical to the one used in the first experiment (Figure 2), except that no adapter stimuli were presented before the test stimuli.

**Figure 4.**
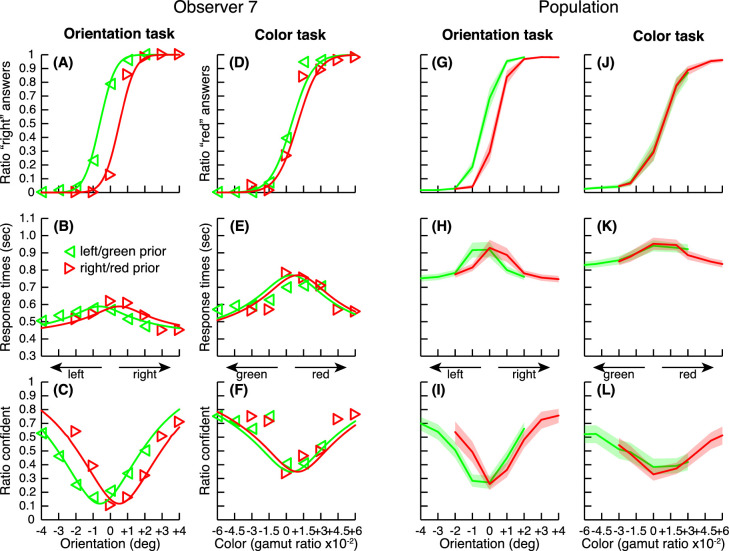
Same as Figure 3 for the second experiment based on a manipulation of the prior probability of occurrence of stimuli. Here, symbols and line colors refer to two different testing ranges of stimuli that were presented in separate blocks of trials. In separate blocks of trials, testing ranges were either in favor of red color or right orientation (red symbols) or in favor of green color or left orientation (green symbols).

Changes in the prior probability of orientation produced a clear bias in the orientation task (see Figure 4). Results presented a striking similarity with the after-effect experiment. The mean normalized adaptations were comparable across three response metrics and tasks. In the orientation task, mean normalized adaptations were 0.62, 0.66, and 0.57 for perceptual decisions, response times, and confidence choices, respectively. In the color task, changes in the prior probability of the stimuli did not produce a corresponding bias. Mean normalized adaptation were 0.08, 0.00, and −0.11 for perceptual decisions, response times, and confidence choices, respectively. Overall, adaptation was significant in the orientation task but not in the color task in a one-tailed *t*-test (see Supplementary Material S4, Table S6), and adaptation strength was not significantly different across metrics in a paired two-tailed *t*-test (see Supplementary Material S4, Tables S7 and S8).

Similarly to the after-effect experiment, we computed correlations among perceptual decisions, response times, and confidence judgments, separately for initial biases (mean PSEs across both prior ranges) and response biases (PSEs for prior ranges). Pairwise correlations between biases were all very high, between 0.6 and 1.0 (Supplementary Material S4, Figure S3 and Tables S9 and S10). The main component in a principal component analysis explained 90% of the variance for the initial biases and 81% for the response biases (93% when analyzing the orientation task alone). Finally a model comparison analysis similar to the one run in the first experiment led, here again, to the same conclusions (see Supplementary Material S5, Figure S4): Modeling biases (either the initial or the response bias) as identical for the three variables (perceptual reports, response times and confidence judgments) accounted for the data just as well as when allowing different biases of the PSEs, responses, or both.

In summary, in this second experiment using unbalanced stimulus statistics, prior probabilities shifted the psychometric, chronometric and confidence functions by equal amounts. Here, again, the distance to the PSE predicts the observers’ confidence, not the actual stimuli. Overall, the effect of prior probabilities seems identical in every respect to the perceptual after-effect. In contrast to the after-effect experiment though, where the adapter was changing from trial to trial, here the effect of prior probabilities needs to be integrated over multiple trials within a block. Therefore, it is remarkable that, in both experiments, response times and confidence tracked so faithfully the perceptual decisions, where these perceptual decisions were biased quickly (at the scale of a single trial in the after-effect experiment) or more slowly (in the prior experiment).

### Confidence is associated with better performance

**Figure 5.**
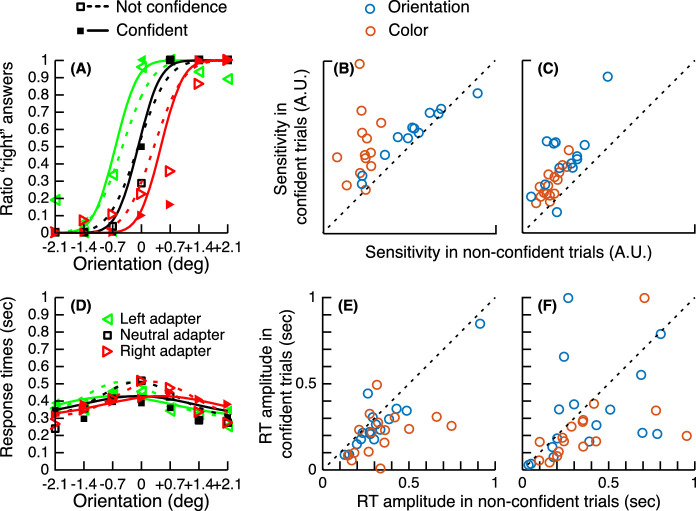
Relationship between confidence judgments and perceptual decisions (sensitivity and response times). (A) Perceptual reports plotted separately for confident (filled markers) and not confident (empty markers) trials and associated psychometric functions (solid and dashed lines) for the orientation task for the same observer as Figure 2. (B) Sensory thresholds in confident trials as a function of sensory thresholds in non-confident trials. Points below the diagonal indicate that observers were better (higher sensitivity) in the confident trials than in the non-confident trials. (C) Same as (B) for the “prior” experiment. (D) Same as (A) for the chronometric functions. (E) Chronometric functions amplitude (maximum response time of the distribution shown in panel D) in confident trials as a function of amplitude in non-confident trials. Points below the diagonal indicate that observers were faster (lower peak response times) in the confident trials than in the non-confident trials. (F) Same as (E) for the “prior” experiment.

High confidence is usually associated with better sensitivity of the observers (Barthelmé & Mamassian, 2009; De Martino, Fleming, Garrett, & Dolan, 2013; Mamassian, 2016; Peirce & Jastrow, 1885) and faster response times (Kiani, Corthell, & Shadlen, 2014; Vickers, 1979). We checked that this was indeed the case in our experiments. In our confidence forced-choice paradigm, participants had to choose which one of two perceptual decisions seemed more likely to be valid. We fitted separate psychometric and chronometric functions (see Methods) for the perceptual decisions that were chosen as the confident ones and for those that were declined in the confidence choice (in this latter case, the observer was more confident in the other perceptual decision of the confidence pair). The slope of the psychometric functions was steeper (higher sensitivity) for the confident trials than for the non-confident trials (see Figures 5A to 5C). Therefore, as expected, if observers had metacognition, confidence was associated with a higher discriminability of the stimulus. We estimated this effect by taking the log-ratio of the sensitivities (ψ) between the confident and non-confident trials. Here, sensitivity (ψ) is the inverse of the standard deviation of the cumulative normal used to fit the data (i.e., 1/σ). This log-ratio was greater than 0, indicating higher sensitivity in the confident trials. This effect was significant for both tasks and both experiments (see Supplementary Material S6, Table S11).

Similarly, we computed the log-ratios of the chronometric function amplitudes *A* between confident and non-confident trials for both the after-effect and prior experiments (Figures 5D to 5F). Here, amplitude *A* describes how much the median response time increased relative to the baseline at the PSE. This log-ratio was smaller than 0, indicating faster responses in the confident trials. This effect was significant (see Supplementary Material S6, Table S11) for both tasks in the after-effect experiment and for the color task in the prior experiment, but not the orientation task (although it did become significant when one outlier was removed).

### Confidence judgments reflect self-consistency

**Figure 6.**
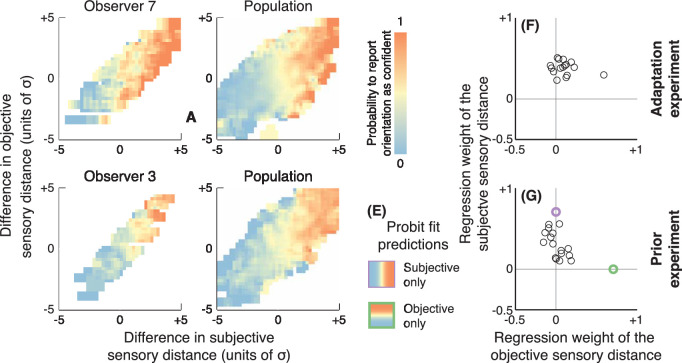
Effects of self-consistency on confidence judgments. (A) The probability to report higher confidence in the orientation than the color decision is plotted as a function of the difference in sensory distances to the subjective criterion (abscissa) and to the objective criterion (ordinates) for the same observer as Figure 2. Sensory distance to the subjective criterion reflects self-consistency, and sensory distance to the objective criterion reflects correctness. (B) Same as (A) averaged across observers. (C, D) Same as (A) and (B) for the prior probability experiment. (E) Two predictions of probit models where observers rely on the subjective sensory distance only (top) or objective sensory distance only (bottom). Regression weights associated with these two examples are plotted for reference in (G), where the purple and green circles represent the subjective and objective models, respectively. (F–G) Probit regression weights of confidence judgments. Observers’ confidence judgments reflect self-consistency (sensory distance to the subjective criterion), not correctness (sensory distance to the objective criterion).

We have shown that the observers’ confidence judgments are consistent with their perceptual reports and response times: They are the least likely to be confident in their answer around the subjective criterion, where they are also slowest. But what information are the observers using to compute their confidence choices? We consider two possible decision variables, one based on the sensory distance of the stimulus to the objective criterion (physical categorical boundary) and the other on the sensory distance to the observer's subjective criterion (the observer's PSE). These objective and subjective sensory distances are in units of sensory noise (i.e., the distance is normalized by the standard deviation of the Gaussian distribution underlying their perceptual psychometric functions; see Equation 9). Objective and subjective distances differ because each observer has some small idiosyncratic initial bias and large biases induced by perceptual adaptation in the first experiment and prior probabilities in the second experiment. We computed the difference in objective sensory distances between each stimulus of a confidence trial, where one stimulus was judged for its color and the other for its orientation, and repeated this for the subjective sensory distances (see Equation 10). Figure 6A plots the probability that one observer (the same as in Figures 3A to 3F) reported being more confident in the orientation decision than the color decision as a function of the difference in objective and subjective sensory distances. Figure 6B shows the average across observers, and Figures 6D and 6E show the same information for the prior probability experiment. In both experiments, confidence judgments were strongly modulated by the subjective sensory distance (abscissa), not the objective sensory distance (ordinate).

To quantify the effect of objective and subjective sensory distances, we fitted a multivariate probit model to the observers’ confidence judgments (see Equation 11 in Methods). The intercept term was not significantly different from 0 in a two-tailed *t*-test in both experiments, *t*(15) = 1.29 and 1.62, *p* = 0.22 and 0.13, indicating that observers did not tend to report either task as more confident. In both experiments, regression weights on the difference in subjective sensory distance were significantly greater than 0 in a one-tailed *t*-test, *t*(15) = 11.59 and 7.51, *p* < 0.001, and higher than regression weights on the difference in objective sensory distance, *t*(15) = 3.75 and 4.97, *p* = 0.002 and *p* < 0.001). However, and importantly, regression weights on the difference in objective sensory distance were not significantly different from 0, *t*(15) = 1.48 and 0.27, *p* = 0.21 and 0.79. In other words, the observers’ confidence judgments were entirely based on the subjective sensory distance of the stimuli (distance to the observer's own PSE, in units of the observer's sensitivity) and not by the objective sensory distance (distance to the physical categorical boundary).

## Discussion

In the present study, we measured how observers adjust their confidence judgments when their percepts fluctuate. In two different experimental paradigms, we induced small controlled perceptual errors while keeping the physical stimuli the same. The two paradigms relied on either after-effects that followed a brief adaptation or on the biases induced by the prior statistics of presented stimuli. These two paradigms can be seen as two different ways to induce a perceptual bias, more low level for the adaptation and more high level for the statistical manipulation. We found that confidence followed perception in the adaptation experiment, which was to be expected if confidence is based on the same sensory evidence that is leading to the perceptual decision (see, for example, Gallagher, Suddendorf, & Arnold, 2019). More surprisingly, we found that confidence also followed perception in the prior manipulation experiment. This result is interesting because we could have thought that observers were biased to respond one way in their perceptual decision but could still sense that their percept was subject to a relatively obvious manipulation of the experimenter (for a study where priors did not always shift confidence judgments, see, for example, Locke, Gaffin-Cahn, Hosseinizaveh, Mamassian, & Landy, 2020). This type of error correction occurs sometimes, such as in the Stroop effect, where participants feel an urge to read a color word rather than reporting the color of the font as instructed (Stroop, 1935), and in these special cases participants can easily detect their errors. This type of error correction did not take place here where perceptual biases were induced by the prior statistics of presented stimuli, indicating that the priors induce genuine changes in what is perceived. In other words, biased percepts induced by prior statistics are qualitatively no different than biased percepts induced by adaptation or correct percepts (those that match the physical stimulus). This effect of the prior statistics is unlikely to be due to potential confounding factors such as adaptation from prior stimuli or sequential response effects, as the observers had to alternate between two different perceptual tasks before providing confidence judgments.

The interpretation of the results of our two experiments is that confidence judgments cannot be used as an internal error signal that our perceptions do not reflect the true state of the world. This is important because the accepted definition of confidence as an estimate of being correct corresponds to a computation relative to an unreachable truth. What matters for the observer is whether she is certain of her perceptual experience, irrespective of objective truth. Instead of being framed as an objective probability on a property of the physical stimulus, we believe that confidence should be appreciated as a subjective reliability of an internal representation of the percept. This change in definition renders the concept of correctness moot. As the notion of subjective correctness is tautological, we propose that confidence is better described as an estimate of the stability of our decision, an estimate of self-consistency. If an observer were asked to judge multiple times the same stimulus, she would be highly confident if she feels that she would give the exact same decision again and again for the same stimulus. Conversely, she would not be confident if she feels her decision was almost random and she could have equally well reached the opposite perceptual decision. This alternative definition of confidence makes testable predictions. For example, confidence should be modulated by the volatility of a percept, or by the number of alternative interpretations. It also fits better with our intuition: When I am looking at a classical visual illusion such the vertical–horizontal illusion, I am reporting that the vertical segment is longer than the horizontal one, and I am confident in what I perceive, even if somebody shows me that I am wrong with a ruler. The importance of self-consistency for confidence was also pointed out by Koriat (for a review, see Koriat, 2011), although in that work, self-consistency was measured across time (an “experience-based” approach). In contrast, we have shown that observers’ sense of confidence tracks perfectly their perceptual reports and response times even when these latter fluctuate from trial to trial, suggesting that an observer's sense of confidence is contained within the current representation of the stimulus (a “direct-access” approach).

Does the refinement of the definition of confidence with reference to self-consistency rather than correctness require that we also change our view on how confidence is computed? One popular model that has been shown to account for accuracy, response times, and confidence judgments is the balance of evidence model (De Martino et al., 2013; Kiani et al., 2014; Mamassian, 2016; Vickers, 1979). In this model, two accumulators representing two perceptual alternatives are racing toward a decision boundary. The first accumulator to reach the boundary wins the decision. This model predicts jointly perceptual decisions, response time (the time at which the boundary is crossed), and confidence as the distance of the losing accumulator to the boundary (the balance of evidence). We have fitted this sequential sampling model to our data (see Supplementary Material S7). We found that this model could reproduce all the effects described in the Results section (see Figures 3 and 4), including higher precision and faster responses in confident trials than non-confident trials. The good match between our results and the model indicates that accuracy, response times, and confidence judgments are generated by a unified mechanism. Put differently, this popular class of models functionally implements something akin to the representation of perceptual stability we have described here, at least in the case where there are only two well defined stimulus categories. At this stage, one can only speculate about the nature of the confidence evidence at the neural level. One possibility for inferring a self-consistency estimate might be to implement a way to resample the sensory evidence using, for example, the bootstrapping procedure (Efron, 1979). In any case, as pointed out by Pouget et al. (2016), finding the neural code for confidence will require a “rigorous computational estimate of confidence.” Our results stress the importance of considering this neural code as an estimate of the stability of the perceptual decision.

In conclusion, we have shown that observers treat identically biased and unbiased perceptual decisions. A unified framework, based on the principle of a balance of evidence, can account for the perceptual decisions, biased or not, the time at which these decisions are taken, and confidence about the uncertainty of these decisions. Therefore, a definition of perceptual confidence that gives prominence to correctness seems misleading. Instead, we have proposed that confidence should be considered as an estimate that one's perceptual decision is self-consistent.

## Supplementary Material

Supplement 1
