# Supplementary material for: Perceptual confidence judgments reflect self-consistency
Source: J Vis. 2021 Nov 18;21(12):8. doi: 10.1167/jov.21.12.8 (PMC8606852; doi:10.1167/jov.21.12.8)
Supplement: Supplement 1 [file jovi-21-12-8_s001.pdf]

SUPPLEMENTARY MATERIALS

Perceptual confidence judgments reflect self-consistency

Baptiste Caziot and Pascal Mamassian

published in *Journal of Vision* in 2021

Supplementary Material S1: Probability of being Self-Consistent

In this section, we present the equation for the probability of being self-consistent as a function of stimulus strength. We consider a perceptual task in which a stimulus has to be categorized as 'Positive' ('P') or 'Negative' ('N'). In our psychophysical experiments, there was a range of stimuli with five different levels of difficulty that we represent by the stimulus strength μ_s.

Because of sensory noise, the observer only has access to some noisy sensory evidence s. We assume that on average the observer has an unbiased estimate of the sensory strength, so the mean of s is μ_s. For simplicity, we further assume that the sensory noise is normally distributed, with common variance σ_s^2 for all stimuli, such that the probability of obtaining sensory evidence s on one trial is

$$\mathrm{P}(s \mid \mu_s) = \varphi(s;\ \mu_s, \sigma_s^2) \ , \tag{E1}$$

where $\varphi(x;\ \mu_s, \sigma_s^2)$ is the probability distribution function of the normal distribution with mean μ_s and variance σ_s^2. In the framework of Signal Detection Theory (Green & Swets, 1966), a perceptual decision (Type 1 decision D) consists in comparing the sensory evidence against a sensory criterion θ_s, namely

$$\begin{cases} D = \text{'P'} & \text{if } s > \theta_s \ , \\ D = \text{'N'} & \text{otherwise} \end{cases} . \tag{E2}$$

What is the probability that the observer's perceptual decision is self-consistent? Here, self-consistent refers to the perceptual decision that matches the most frequent decision for a particular stimulus μ_s. If we call *M* this most frequent decision, we have

$$M(\mu_s) = \begin{cases} \text{'P'} & \text{if } \mu_s > \theta_s \ , \\ \text{'N'} & otherwise \end{cases} \tag{E3}$$

We can now look at the probability that the observer's perceptual decision is self-consistent for a given displayed stimulus μ_s

$$P(\text{self-consistent} \mid \mu_s) = \int_{-\infty}^{+\infty} P(\text{self-consistent} \mid \mu_s, s)\ \mathrm{P}(s \mid \mu_s)\, ds$$

$$= \int_{-\infty}^{+\infty} P(D = M(\mu_s) \mid \mu_s, s)\ \mathrm{P}(s \mid \mu_s)\, ds$$

$$= \begin{cases} \int_{-\infty}^{+\infty} P(D = \text{'P'} \mid s)\ \mathrm{P}(s \mid \mu_s)\, ds & \text{if } \mu_s > \theta_s\,, \\ \int_{-\infty}^{+\infty} P(D = \text{'N'} \mid s)\ \mathrm{P}(s \mid \mu_s)\, ds & otherwise \end{cases} \tag{E4}$$

$$= \begin{cases} \int_{\theta_s}^{+\infty} \varphi(s;\ \mu_s, \sigma_s^2)\, ds = \Phi((\mu_s - \theta_s)/\sigma_s) & \text{if } \mu_s > \theta_s\,, \\ \int_{-\infty}^{\theta_s} \varphi(s;\ \mu_s, \sigma_s^2)\, ds = 1 - \Phi((\mu_s - \theta_s)/\sigma_s) & otherwise \end{cases}$$

where Φ is the cumulative of the standard normal distribution. In short, we obtain

$$P(\text{self-consistent} \mid \mu_s) = \Phi(|\,\mu_s - \theta_s| \,/\, \sigma_s) \tag{E5}$$

If instead of self-consistency we were interested in correctness, then we would have to replace the condition $(\mu_s > \theta_s)$ by $(\mu_s > 0)$ in Equation E3, so that

$$P(\text{correct} \mid \mu_s) = \begin{cases} \Phi((\mu_s - \theta_s)/\sigma_s) & \text{if } \mu_s > 0\,, \\ 1 - \Phi((\mu_s - \theta_s)/\sigma_s) & otherwise \end{cases}\ . \tag{E6}$$

Instead of focusing on the probability that the observer's perceptual decision is self-consistent for a given displayed stimulus μ_s, we can also look at self-consistency on each single trial. The probability that the observer's perceptual decision is self-consistent, given that she has access to sensory evidence s is

$$P(\text{self-consistent} \mid s) = \sum_{\mu_s} \left(P(\text{self-consistent} \mid \mu_s, s)\ \mathrm{P}(\mu_s \mid s) \right)$$

$$= \sum_{\mu_s} \left(P(D = M(\mu_s) \mid \mu_s, s)\ \mathrm{P}(\mu_s \mid s) \right)$$

$$= \sum_{\mu_s > \theta_s} \left(P(D = \text{'P'} \mid s)\ \mathrm{P}(\mu_s \mid s) \right) + \sum_{\mu_s \le \theta_s} \left(P(D = \text{'N'} \mid s)\ \mathrm{P}(\mu_s \mid s) \right) \tag{E7}$$

$$= \begin{cases} \sum_{\mu_s > \theta_s} \mathrm{P}(\mu_s \mid s) & \text{if } s > \theta_s\,, \\ \sum_{\mu_s \le \theta_s} \mathrm{P}(\mu_s \mid s) & otherwise \end{cases}$$

Using Bayes' rule, we have

$$\mathrm{P}(\,\mu_s \mid s\,) = \mathrm{P}(s \mid \mu_s)\,\mathrm{P}(\mu_s)/\,\mathrm{P}(s) = \frac{\varphi(s;\,\mu_s,\sigma_s^2)\,\mathrm{P}(\mu_s)}{\sum_{\mu_s}\left(\varphi(s;\,\mu_s,\sigma_s^2)\,\mathrm{P}(\mu_s)\right)}\;, \tag{E8}$$

so that Equation E7 can be rewritten as

$$P(\text{self-consistent} \mid s) = \begin{cases} \dfrac{\sum_{\mu_s>\theta_s}\left(\varphi(s;\,\mu_s,\sigma_s^2)\,\mathrm{P}(\mu_s)\right)}{\sum_{\mu_s}\left(\varphi(s;\,\mu_s,\sigma_s^2)\,\mathrm{P}(\mu_s)\right)} & \text{if } s>\theta_s\,, \\ \\ \dfrac{\sum_{\mu_s\le\theta_s}\left(\varphi(s;\,\mu_s,\sigma_s^2)\,\mathrm{P}(\mu_s)\right)}{\sum_{\mu_s}\left(\varphi(s;\,\mu_s,\sigma_s^2)\,\mathrm{P}(\mu_s)\right)} & otherwise \end{cases}\;. \tag{E9}$$

Figure 1 in the main text shows the probability of being self-consistent as a function of sensory evidence when there are five possible stimuli with varying strengths that can occur with equal probability.

There is one common special case where Equation E7 is simplified. When a stimulus can have any strength, and when all of these strengths have an equal probability of occurrence, then this equation becomes

$$P(\text{self-consistent} \mid s) \;=\; \begin{cases} \Phi((s-\theta_s)/\sigma_s) & \text{if } s>\theta_s \\ \\ 1-\Phi((s-\theta_s)/\sigma_s) & \text{otherwise} \end{cases}\;, \tag{E10}$$

or in short

$$P(\text{self-consistent} \mid s) \;=\; \Phi(\mid (s-\theta_s)/\sigma_s \mid)\;\;. \tag{E11}$$

Supplementary Material S2: Methodological detail for Experiment 2

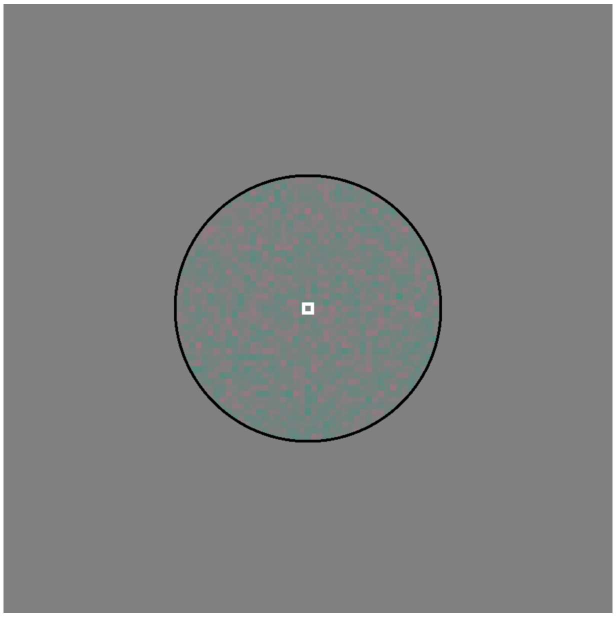

Figure S1: Example of color stimulus used in Experiment 2.

Supplementary Material S3: Correlations of initial biases and adaptation amplitude in Experiment 1

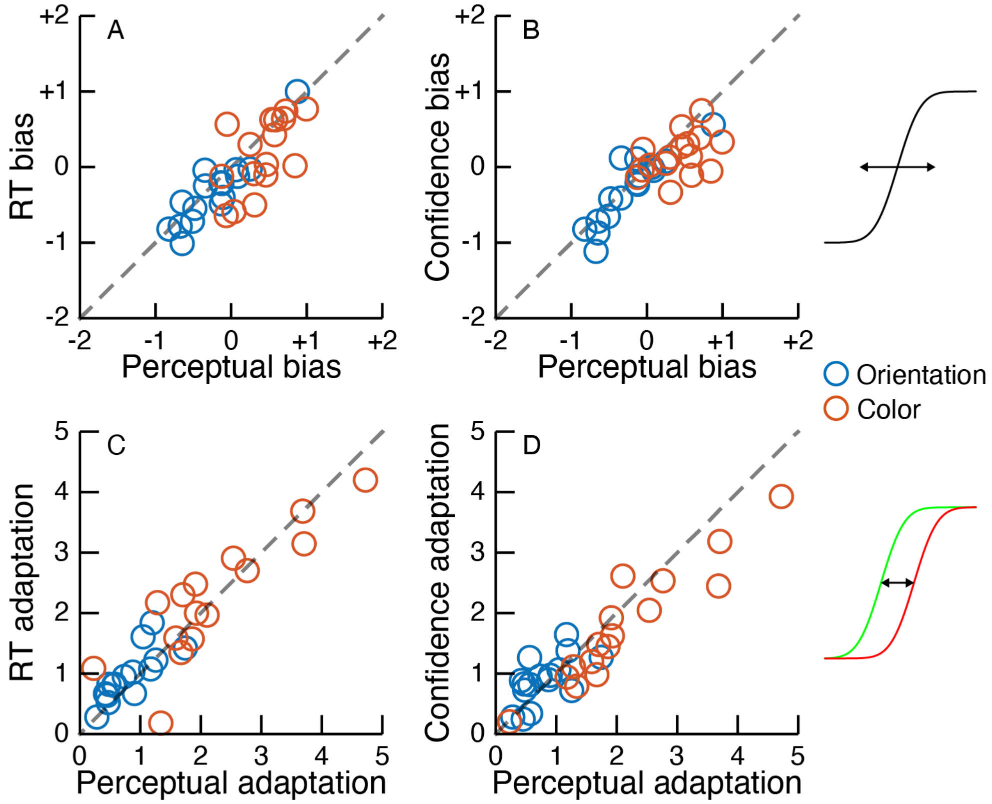

Figure S2: A: Normalized initial bias estimated from the RT curves plotted as a function of the initial bias estimated from the psychometric functions for each observer (circles) and task (blue for orientation and red for color) separately in Experiment 1. B: Initial bias estimated from the confidence curves plotted as a function of the initial bias estimated from the psychometric functions. C: Amplitude of perceptual adaptation estimated from the RT curves plotted as a function of the amplitude of adaptation estimated from the psychometric functions. D: Amplitude of perceptual adaptation estimated from the confidence curves plotted as a function of the amplitude of adaptation estimated from the psychometric functions.

Metric	Orientation		Color	
	t(15)	p	t(15)	p
PSE	7.99	<0.001	7.62	<0.001
RT peak	9.30	<0.001	6.12	<0.001
Confidence trough	9.15	<0.001	7.31	<0.001

Table S1: T-tests on normalized adaptation parameters in the after-effect experiment.

	PF	RT	Conf
PF	0 (1)	1.08 (0.29)	0.70 (0.49)
RT	1.08 (0.29)	0 (1)	0.69 (0.40)
Conf	0.70 (0.49)	0.69 (0.40)	0 (1)

Table S2: Pairwise t-tests on normalized adaptation parameters for the orientation task in the after-effect experiment (t(15) and p-value).

	PF	RT	Conf
PF	0 (1)	0.30 (0.77)	0.97 (0.34)
RT	0.30 (0.77)	0 (1)	0.57 (0.57)
Conf	0.97 (0.34)	0.57 (0.57)	0 (1)

Table S3: Pairwise t-tests on normalized adaptation parameters for the color task in the after-effect experiment (t(15) and p-value).

	PF	RT	Conf
PF	1	0.82	0.82
RT	0.82	1	0.77
Conf	0.82	0.77	1

Table S4: Correlation between initial bias estimated from psychometric functions, RT curves and confidence curves for Experiment 1.

	PF	RT	Conf
PF	1	0.84	0.93
RT	0.84	1	0.82
Conf	0.93	0.82	1

Table S5: Correlation between adaptation amplitude estimated from psychometric functions, RT curves and confidence curves for Experiment 1.

Supplementary Material S4: Correlations of initial biases and adaptation amplitude in Experiment 2

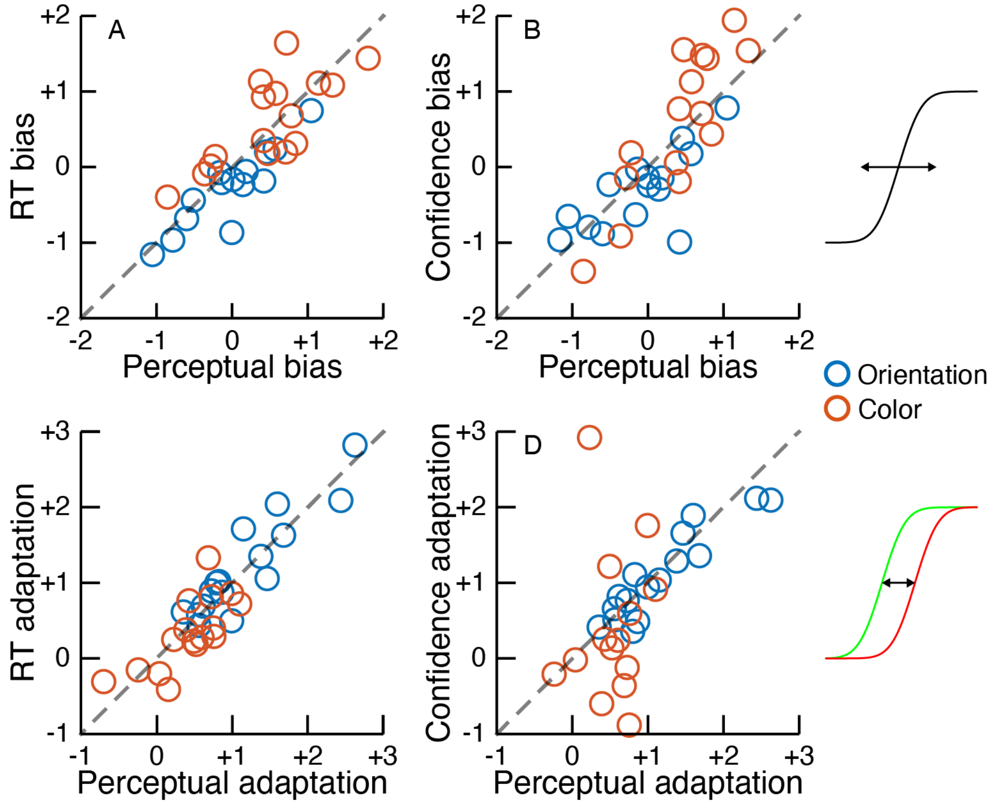

Figure S3: Same as Figure S2 but for Experiment 2.

Metric	Orientation		Color	
	t(15)	p	t(15)	p
PSE	5.02	<0.001	0.87	0.40
RT peak	5.12	<0.001	0.05	0.96
Confidence trough	5.16	<0.001	0.53	0.60

Table S6: T-tests on normalized adaptation parameters in the response bias experiment.

	PF	RT	Conf
PF	0 (1)	1.10 (0.28)	0.77 (0.45)
RT	1.10 (0.28)	0 (1)	0.93 (0.36)
Conf	0.77 (0.45)	0.93 (0.36)	0 (1)

Table S7: Pairwise t-tests on normalized adaptation parameters in response bias experiment (t(15) and p-value).

	PF	RT	Conf
PF	0 (1)	0.28 (0.78)	0.00 (1.00)
RT	0.28 (0.78)	0 (1)	0.20 (0.85)
Conf	0.00 (1.00)	0.20 (0.85)	0 (1)

Table S8: Pairwise t-tests on normalized adaptation parameters in response bias experiment (t(15) and p-value).

	PF	RT	Conf
PF	1	0.90	0.87
RT	0.90	1	0.81
Conf	0.87	0.81	1

Table S9: Correlation between initial bias estimated from psychometric functions, RT curves and confidence curves for Experiment 2.

	PF	RT	Conf
PF	1	0.90	0.66
RT	0.90	1	0.62
Conf	0.66	0.62	1

Table S10: Correlation between adaptation amplitude estimated from psychometric functions, RT curves and confidence curves for Experiment 2.

Supplementary Material S5: Model comparison analysis

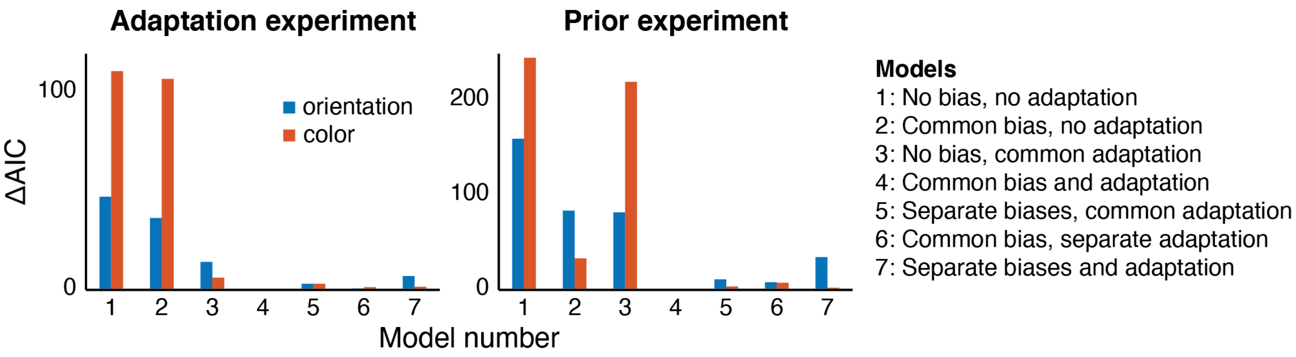

Figure S4: Mean difference in AIC scores between different models (1-7) and model 4 (common bias and adaptation). For both tasks and both experiments, the model that received the lowest AIC score assumed a common initial bias and adaptation parameter for all 3 metrics (perceptual reports, RTs and confidence judgments).

Supplementary Material S6: Relationship between confidence and performance

	After-effect experiment				Prior experiment			
Metric	Orientation		Color		Orientation		Color	
	t(15)	p	t(15)	p	t(15) [t(14)]	p	t(15)	p
$log\left(\frac{\psi_{confident}}{\psi_{not-confident}}\right)$	3.95	0.001	6.19	<0.001	5.39	<0.001	9.21	<0.001
$log\left(\frac{A_{confident}}{A_{not-confident}}\right)$	3.21	0.006	4.40	<0.001	1.71 [2.86]	0.11 [0.01]	4.23	<0.001

Table S11: T-tests on normalized adaptation parameters in the response bias experiment. Numbers between brackets show results when one outlier was removed from the analysis.

Supplementary Material S7: Accumulator model

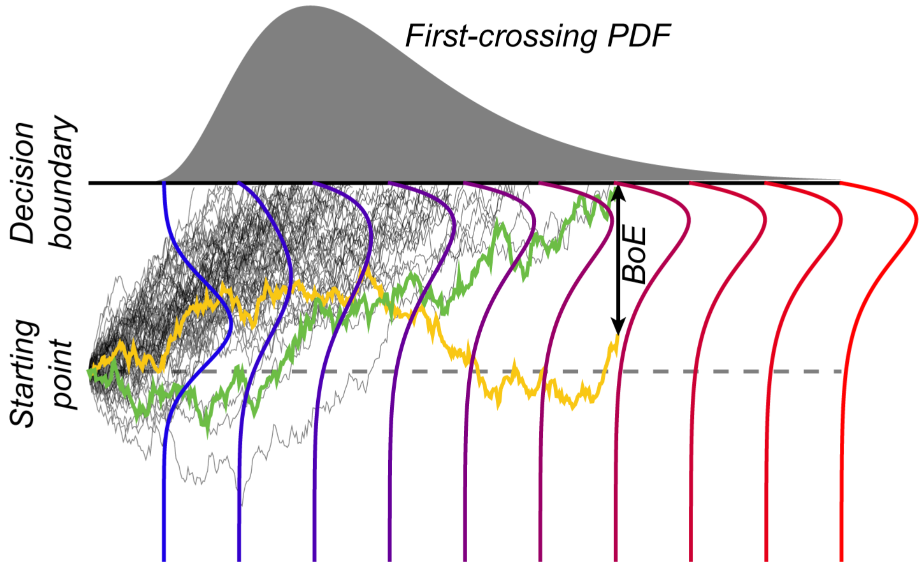

Figure S5: Depiction of the sequential sampling model. Two accumulators (green and yellow lines) compete to reach a decision boundary first. When one accumulator reaches the decision boundary, the observer commits to a perceptual decision associated with this accumulator (here green). Over successive trials the accumulators produce a first-crossing probability density function as depicted on top (gray, equation E13). Confidence is implemented as a Balance of Evidence, that is the distance between the winning and the losing accumulator. Blue to red lines are the probability density functions of one of the accumulator (here the green one) at different times (equation E18).

The accumulated signal is generated by a Wiener process with drift μ and variance σ^2

$$dx = \mu dt + \sigma dW \tag{E12}$$

Therefore the probability for an accumulator to reach the decision bound z at a time t follows an inverse Gaussian distribution (2) with probability f and cumulative F density functions

$$f(t|z,\mu,\sigma) = \frac{z}{\sigma\sqrt{2\pi t^3}} e^{-\frac{(z-\mu t)^2}{2\sigma^2 t}} \tag{E13}$$

$$F(t|z,\mu,\sigma) = \Phi\left(+\frac{z}{\sigma\sqrt{t}}\left(\frac{\mu t}{z} - 1\right)\right) + e^{\frac{2\mu z}{\sigma^2}} \Phi\left(-\frac{z}{\sigma\sqrt{t}}\left(\frac{\mu t}{z} + 1\right)\right) \tag{E14}$$

where Φ is the normal *CDF*.

The probability that the accumulator A wins the decision is thus given by the probability that the accumulator A reaches the boundary z at time t while the accumulator B has not yet reached it

$$p(resp = A) = \int_0^{+\infty} f(t|z,\mu_A,\sigma)\big(1 - F(t|z,\mu_B,\sigma)\big)dt \tag{E15}$$

The mean response time is

$$\overline{RT} = \int_0^{+\infty} t.f_{tot}(t)dt \tag{E16}$$

with $f_{tot}(t)$ the distribution of response times for both accumulators

$$f_{tot} = f(t|z,\mu_A,\sigma)\big(1 - F(t|z,\mu_B,\sigma)\big) + f(t|z,\mu_B,\sigma)\big(1 - F(t|z,\mu_A,\sigma)\big) \tag{E17}$$

The probability that an accumulator is at *x* at time *t* is $p(x,t) = \mathcal{N}\big(x,\mu t,\sigma\sqrt{t}\big)$, but that distribution includes paths where the accumulator passed the decision bound and went back under it at later times. Because of the statistical properties of the Wiener process, the probability of such a path is the same as the one of a mirror accumulator following a path symmetrical about the decision bound (but with opposite drift). Thus in the probability that the accumulator is at *x* is the sum of a Gaussian and an anti-Gaussian, and can be rewritten as (Redner, 2001)

$$p(x,t,\mu) = \frac{1}{\sigma\sqrt{2\pi t}} e^{-\frac{(x-z-\mu t)^2}{2\sigma^2 t}} \left(1 - e^{-\frac{2xz}{\sigma^2 t}}\right) \tag{E18}$$

which can be used to derive the cumulative density function

$$\begin{aligned} p(x < X|t) &= \int_0^X p(x,t)dx = \int_0^X \frac{1}{\sqrt{2\pi\sigma^2 t}} e^{-\frac{(x-z-\mu t)^2}{2\sigma^2 t}} \left(1 - e^{-\frac{2xz}{\sigma^2 t}}\right) dx \\ &= \int_0^X \frac{1}{\sqrt{2\pi\sigma^2 t}} \left(e^{-\frac{(x-z-\mu t)^2}{2\sigma^2 t}} - e^{-\frac{2z\mu}{\sigma^2}} e^{-\frac{(x+z-\mu t)^2}{2\sigma^2 t}}\right) dx \end{aligned} \tag{E19}$$

Substituting $u^2 = \frac{(x-z-\mu t)^2}{2\sigma^2 t}$ and $v^2 = \frac{(x+z-\mu t)^2}{2\sigma^2 t}$, we get

$$\begin{aligned} p(x < X|t) &= \frac{1}{\sqrt{2\pi\sigma^2 t}} \left[\int_{\frac{-z-\mu t}{2\sigma^2 t}}^{\frac{X-z-\mu t}{2\sigma^2 t}} e^{-u^2} du - e^{-\frac{2z\mu}{\sigma^2}} \int_{\frac{+z-\mu t}{2\sigma^2 t}}^{\frac{X+z-\mu t}{2\sigma^2 t}} e^{-v^2} dv\right] \\ &= \frac{1}{2\sqrt{2\sigma^2 t}} \frac{2}{\sqrt{\pi}} \Bigg[\int_0^{\frac{X-z-\mu t}{2\sigma^2 t}} e^{-u^2} du - \int_0^{\frac{-z-\mu t}{2\sigma^2 t}} e^{-u^2} du \\ &\quad - e^{-\frac{2z\mu}{\sigma^2}} \left(\int_0^{\frac{X+z-\mu t}{2\sigma^2 t}} e^{-v^2} dv - \int_0^{\frac{+z-\mu t}{2\sigma^2 t}} e^{-v^2} dv\right)\Bigg] \\ &= \frac{1}{2\sqrt{2\sigma^2 t}} \Bigg[erf\left(\frac{X-z-\mu t}{2\sigma^2 t}\right) - erf\left(\frac{-z-\mu t}{2\sigma^2 t}\right) \\ &\quad - e^{-\frac{2z\mu}{\sigma^2}} \left(erf\left(\frac{X+z-\mu t}{2\sigma^2 t}\right) - erf\left(\frac{+z-\mu t}{2\sigma^2 t}\right)\right)\Bigg] \end{aligned} \tag{E20}$$

Integrating the product of $p(x,t)$ and $p(x < X|t)$ for 2 different decisions across distance and time gives the probability that confidence is higher for the first decision.

REFERENCES

Green, D. M & Swets, J.A. (1966). *Signal Detection Theory and Psychophysics.* Wiley.

Redner, S. (2001). *A Guide to First-Passage Processes.* Cambridge University Press.
